# Comparison of programmed death‐ligand 1 expression in adenocarcinoma and squamous cell carcinoma of the cervix in paraffin blocks of patients with cervical cancer

**DOI:** 10.1002/cnr2.2057

**Published:** 2024-04-25

**Authors:** Maryam Sadat Hosseini, Fatemeh Shafizadeh, Mohammad Hashemi Bahremani, Farah Farzaneh, Tahereh Ashrafganjoei, Maliheh Arab, Maryam Talayeh, Fatemeh Jafari, Alireza Abdshah

**Affiliations:** ^1^ Preventative Gynecology Research Center, Department of Obstetrics and Gynecology, Imam Hossein Hospital Shahid Beheshti University of Medical Sciences Tehran Iran; ^2^ Department of Gynecology Oncology, Imam Hossein Medical Center Shahid Beheshti University of Medical Sciences Tehran Iran; ^3^ Department of Pathology, Imam Hossein Medical Center Shahid Beheshti University of Medical Sciences Tehran Iran; ^4^ Radiation Oncology Research Center (RORC), Imam Khomeini Hospital Complex Tehran University of Medical Sciences Tehran Iran; ^5^ Radiation Oncology Department, Cancer Institute, Imam‐Khomeini Hospital Complex Tehran University of Medical Sciences Tehran Iran; ^6^ School of Medicine Tehran University of Medical Sciences Tehran Iran

**Keywords:** adenocarcinoma, cervical cancer, immunohistochemistry, PD‐1, PD‐L1, protein expression, squamous cell carcinoma

## Abstract

**Aims:**

Cervical cancer (CC) is a common malignancy in women, predominantly caused by human papillomavirus. The most subtypes are adenocarcinomas (AC) and squamous cell carcinomas (SCC), which show various features and treatment responses. Programmed death‐ligand 1 (PD‐L1) and programmed cell death protein 1 (PD‐1) as Immune checkpoint molecules, play a role in immune evasion. We investigated PD‐L1 expression in AC and SCC of the cervix and explored its link to clinical characteristics.

**Methods and results:**

The present cross‐sectional research was done between 2016 and 2022 on samples in Shahid Beheshti University of Medical Sciences‐affiliated hospitals in Iran. Histological tissue samples of CCs (16 AC and 48 SCC) were assessed, and clinical information was obtained by reviewing their medical documents. PD‐L1 expression was evaluated by immunohistochemistry and we used the combined positive score. SCC cases showed a higher (not significant) PD‐L1 expression. The PD‐L1 expression and clinical characteristics were not significantly correlated in both subgroups.

**Conclusion:**

Although SCC cases exhibited higher PD‐L1 expression, this difference was non‐significant. More investigations should highlight the role of PD‐L1 in CC and the potential benefits of immunotherapy.

## 1 INTRODUCTION

Cervical cancer (CC) ranks fourth of all cancers affecting women caused by infection with the high‐risk human papillomavirus (HPV) strains, most commonly HPV18 and HPV16.^1^ There are two main types, adenocarcinomas (AC) and squamous cell carcinoma (SCC). Specifically, 70%–75% of the CC cases in the US are of squamous cell carcinoma histology and 25% are adenocarcinomas (including adenosquamous).^2^, ^3^, ^4^

CC can easily be screened (by a simple and effective smear), diagnosed, and treated in its early stages; however, if delayed, the treatment can be difficult and unsuccessful. Tissue sampling from such smears, in conjunction with medical imaging, guides the diagnosis of cancer.^5^ In communities with active screening programs, the mortality from CC has decreased by 50% over the past 30 years.^6^

CC is managed with fertility preservation surgeries, extra fascial hysterectomy, pelvic lymphadenectomy, radiotherapy, and/or chemotherapy, depending on the stage and size.^7^ Recently, the number of patients with adenocarcinoma has increased. This histology is associated with poorer survival compared to squamous cell carcinoma, particularly in the more advanced stages such as lymph‐node‐positive tumors. Novel treatments based on immunotherapy are being developed with the hope of improving CC prognosis.^4^, ^8^

Programmed death‐ligand 1 (PD‐L1) protein (B7‐H1 or CD274) is encoded by the CD274 gene. This protein inhibits T cells through binding to the programmed cell death protein 1 (PD‐1) receptor. The PD‐1/PD‐L1 pathway in the immune system prevents the excessive activity of T and B lymphocytes as a negative regulatory mechanism.^9^ Cancer cells with high expression of PD‐L1 and through this ligand interaction with the PD‐1 receptor on the T lymphocyte surface escape from the immune system and suppress the cellular response against the tumor. When PD‐L1 on a tumor cell can attach to PD‐1 on a T cell, and create an inhibitory signal, which can lead to T cell destruction. This process regulates the immune system and health, but it can also reduce the activity of the immune system against cancer. Modern therapies that target immune checkpoint molecules, like the PD‐1, inhibit the immunosuppressive ability of the tumor leading to promising long‐term clinical response.^10^, ^11^, ^12^, ^13^, ^14^

A noteworthy proportion of CC samples have been observed to express PD‐L1.^15^ Thus, PD‐L1 plays a key role in the immune evasion of CC by targeting the PD‐L1/PD‐1 pathway.^16^ The PD‐L1 and PD‐1 expression had a positive correlation with cervical intraepithelial neoplasia (CIN) and tumor metastasis progression, and therefore are applied as prognostic biomarkers to assess CIN.^17^ However, there is no consensus regarding the pattern of PD‐L1 expression among different malignancies of the cervix. Most of the current literature is also based on other populations.^18^, ^19^ As such, we decided to compare the PD‐L1 expression in CC of SCC and AC main histological subtypes and examined its correlation with pathological as well as clinical features in an Iranian population.

## 2 MATERIALS AND METHODS

We conducted this cross‐sectional study between 2016 and 2022, in hospitals of Shahid Beheshti University of Medical Sciences in Iran.

### 2.1 Study procedure

All paraffin block specimens of histologically diagnosed CCs (SCC and AC) were evaluated in the pathology department of hospitals between 2016 and 2022. The demographics and clinical information of CC patients were extracted from their medical documents. All CC cases diagnosed during the research period (2016–2022) were included. Patients whose paraffin blocks did not exist or did not have enough diagnostic tissue were excluded from this research.

We performed immunohistochemistry as instructed. We utilized polyclonal antibodies for PD‐L1, plus hematoxylin and eosin staining, while we used tonsillar tissue blocks as control blocks for PD‐L1 IHC positivity and negativity.

We employed the combined positive score (CPS) to categorize the expression PD‐L1 as a binary variable. CPS is calculated by determining the count of lymphocytes, positive malignant cells, and macrophages (determined by partial and/or complete peripheral linear plasma membrane staining) divided by the count of viable tumor cells ×100. CPS larger or equal to 1% was considered a positive PD‐L1 expression in CC, and CPS less than 1% was regarded as a negative PD‐L1 expression.^16^ We then compared the PD‐L1 protein expression levels between two SCC and AC groups.

### 2.2 Sampling method

The sample size was calculated by referring to the current literature^17^ and using the software g*power (version 3). We concluded that a sample size of 40, with alpha 0.05 and power 0.8, would be sufficient. After checking with our sample database of all patients from 2016 to 2022, we had data on 64 patients available, and we included all the patients from our records in this study. Accordingly, all paraffin block samples of histologically diagnosed CCs (SCC and AC) in the pathology departments from 2016 to 2022 were sampled in the study.

### 2.3 Data analysis

All data were entered into and analyzed by SPSS 26. We performed a *t* test (or non‐parametric Mann–Whitney *U*) and Chi‐square (or Fisher's exact test) to compare the distribution of respectively continuous and categorical variables. A significance level of less than 0.05 was regarded.

## 3 RESULTS

Paraffin block samples of histologically diagnosed CC were examined in 48 patients with SCC and 16 patients with AC.

A comparison of clinical characteristics of the patients between the two groups of SCC and AC did not reveal any significant difference. The comparison is summarized in Table 1. The tumor size ≥4 cm was observed larger in cases of the AC group (*p* = .47) and the mean tumor size was greater in the SCC group in comparison to the AC group (*p* = .53), albeit not statistically significant. In the SCC group, in almost half of the cases, parametrial and lymph node involvement were observed; nonetheless, no significant differences were detected in comparison to the AC group.

**TABLE 1:** Comparison of clinical characteristics in 48 patients with squamous cell carcinomas (SCC) and 16 patients with adenocarcinomas (AC).

Variable		Squamous cell carcinoma *N* = 48, *N* (%)	Adenocarcinoma *N* = 16, *N* (%)	*p* value	Test
Age	Years	49.5 (SD = 10.2)	50.4 (SD = 14.5)	.9	Mann–Whitney *U*
Menopause	Menopause	25 (52.1 %)	8 (50 %)	.88	Chi‐square
	Pre‐menopause	23 (47.9 %)	8 (50 %)		
Tumor size (cm)	<4	25 (52.1 %)	5 (31.3 %)	.148	Chi‐square
	≥4	23 (47.9 %)	11 (68.7 %)		
Tumor size (cm)	Mean (SD)	4.58 (1.93)	4.21 (2.04)	.519	*t* test
Parametrial involvement	Yes	27 (56.2 %)	5 (31.2 %)	.083	Chi‐square
	No	21 (43.8 %)	11 (68.8 %)		
Lymph node involvement	Yes	21 (43.8 %)	7 (43.8 %)	1	Chi‐square
	No	27 (56.2 %)	9 (56.2 %)		

The mean PD‐L1 expression in the SCC group was 5.19 (SD = 7.1), while it was 3.44 (SD = 5.7) (*p* = .37) in the AC group. There were more positive cases in the SCC group with expressions of the protein, though this difference was not significant (*p* = .31) (Table 2).

**TABLE 2:** Comparison of mean expression of programmed death‐ligand 1 (PD‐L1) in paraffin block samples of histologically diagnosed cervical cancers in 48 patients with squamous cell carcinomas (SCC) and 16 patients with adenocarcinomas (AC).

PD‐L1	Squamous cell carcinoma, *N* = 48	Adenocarcinoma, *N* = 16	*p* value	Test
Mean (SD)	5.19 (7.11)	3.44 (5.69)	0.33	Mann–Whitney *U*
Yes, *N* (%)	25 (52.1%)	6 (37.5%)	0.31	Chi‐square
No, *N* (%)	23 (47.9%)	10 (62.5%)		

Considering PD‐L1 as a qualitative measure (positive or negative), no significant correlation was recorded between age, menopause, FIGO staging <I B2, tumor size, parametrial involvement, as well as lymph node involvement with PD‐L1 in AC and SCC groups (Table 3).

**TABLE 3:** Association of programmed death‐ligand 1 (PD‐L1) expression (positive or negative) with patients' clinical characteristics in squamous cell carcinomas (SCC) and adenocarcinomas (AC) groups.

Variable		Groups	Negative PD‐L1 (*n* = 33)	Positive PD‐L‐1(*n* = 31)	*p* value	Test
*Age* Mean (SD)	Years	SCC	47.74 (9.3)	51.16 (10.8)	.25	*t* test
		AC	50.40 (14.75)	50.3 (15.58)	.99	*t* test
*Menopause* *N* (%)	Menopause	SCC	10 (43.5%)	15 (60%)	.25	Chi‐square
	Pre‐menopause		13 (56.5%)	10 (40%)		
	Menopause	AC	5 (50%)	3 (50%)	1	Chi‐square
	Pre‐menopause		5 (50%)	3 (50%)		
*FIGO staging* <I B2 *N* (%)	Yes	SCC	1 (5.9%)	4 (19%)	.35	Fisher's exact
	No		16 (94.1%)	17 (81%)		
	Yes	AC	2 (25%)	1 (16.7%)	1	Fisher's exact
	No		6 (75%)	5 (83.3%)		
Tumor size Mean (SD)	Centimeter	SCC	4.64 (1.73)	4.52 (2.13)	.83	*t* test
		AC	4.35 (2.37)	3.98 (1.53)	.72	Mann–Whitney *U*
Tumor size	<4 cm	SCC	12 (52.2%)	13 (52%)	.99	Chi‐square
	≥4 cm		11 (47.8%)	12 (48%)		
	<4 cm	AC	4 (40%)	1 (16.7%)	.58	Fisher's exact
	≥4 cm		6 (60%)	5 (83.3%)		
*Parametrial involvement* *N* (%)	Yes	SCC	16 (69.6%)	11 (44%)	.07	Chi‐square
	No		7 (30.4%)	14 (56%)		
	Yes	AC	4 (40%)	1 (16.7%)	.58	Fisher's exact
	No		6 (60%)	5 (83.3%)		
*Lymph node involvement* *N* (%)	Yes	SCC	12 (52.2%)	9 (36%)	.26	Chi‐square
	No		11 (47.8%)	16 (64%)		
	Yes	AC	5 (50%)	2 (33.3%)	.63	Fisher's exact
	No		5 (50%)	4 (66.7%)		

*Note*: If combined positive score (CPS) ≥1%, it was considered as positive PD‐L1 expression in cervical cancer. If CPS less than 1%, it was considered as negative PD‐L1 expression.

In Table 4, PD‐L1 values are quantitatively compared. The findings indicate that no significant correlation was recorded between menopause, FIGO staging <I B2, tumor size <4 cm, parametrial involvement, and lymph node involvement.

**TABLE 4:** Association of mean expression of programmed death‐ligand 1 (PD‐L1) with patients' clinical characteristics in squamous cell carcinomas (SCC) and adenocarcinomas (AC) groups.

		Groups	PD‐L1	*p* value	Test
Variable			Mean (SD)		
Menopause	Post‐menopause	SCC	5.67 (7.73)	.47	Mann–Whitney *U* test
	Pre‐menopause		4.41 (5.83)		
	Post‐menopause	AC	3.57 (4.76)	.96	
	Pre‐menopause		4.29 (7.32)		
FIGO staging < I B2	Yes	SCC	9 (7.42)	.15	
	No		4.52 (6.73)		
	Yes	AC	6.67 (11.55)	1	
	No		3.18 (4.05)		
Tumor size	<4 cm	SCC	5.83 (8.23)	.64	
	≥4 cm		4.45 (5.56)		
	<4 cm	AC	4 (8.94)	.66	
	≥4 cm		3.89 (4.17)		
Parametrial involvement	Yes	SCC	3.05 (5.18)	.05	
	No		7.94 (8.05)		
	Yes	AC	2.5 (5)	.51	
	No		4.5 (6.43)		
Lymph node involvement	Yes	SCC	4.94 (8.38)	.26	
	No		5.25 (5.44)		
	Yes	AC	3 (4.47)	.61	
	No		4.44 (6.82)		

**FIGURE 1:**
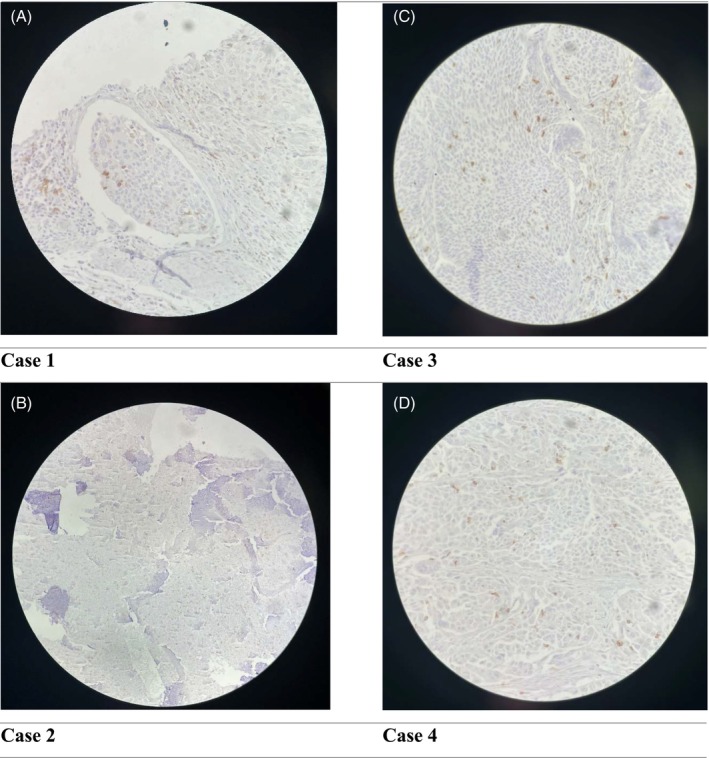
Correlation of mean expression of programmed death‐ligand 1 (PD‐L1) with different variables in squamous cell carcinomas (SCC) and adenocarcinomas (AC) groups. Programmed death‐ligand 1 expression in cervical cancer. Various CPS of PD‐L1 expression (in brown) were detected in SCC and AC. (A) Case 1: PD‐L1 positive in SCC with expression CPS >1% in tumor cells. (B) Case 2: PD‐L1 expression is negative in SCC. (C) Case 3: PD‐L1 positive in SCC with CPS > 1% expression in tumor cells and expression in inflammatory cells. (D) Case 4: PD‐L1 expression in AC with CPS >1% in tumor cells.

The immunohistochemically prepared slides were visualized under a bright‐field microscope, and researchers assessed the CPS. This score was regarded as the percentage of PD‐L1‐stained cells (macrophages, tumor cells and lymphocytes) in relation to the total count of viable tumor cells, multiplied by 100 (Figure 1). Tumor fields were distinguishable from healthy tissue using nuclear hematoxylin staining. Tumor cells were considered positive for PD‐L1 if over 1% of the tumor cells exhibited PD‐L1 positivity. Scores were assigned based on the existence of PD‐L1‐positive tumor‐infiltrating cells (No/Yes) and the accumulation of immune cells surrounding tumor areas producing a PD‐L1‐positive cordon (No/Yes). Histiocytes and stromal cells in B‐cell follicles served as an internal control for PD‐L1 positivity.

## 4 DISCUSSION

We analyzed the PD‐L1 protein expression in AC and SCC of the cervix. Although the the SCC group showed a higher PD‐L1 expression than the AC group, this disparity was non‐significant. This observation aligns with findings from prior research. For instance, Heeren et al.^16^ conducted a similar investigation evaluating PD‐L1 expression in pathology specimens from two patient groups with SCC and AC. Their results indicated that the SCC subtype exhibited more positive PD‐L1 expression and contained a greater number of PD‐L1‐positive macrophages compared to AC (*p* < .001). Furthermore, this research showed lower disease‐free survival (DFS) and overall survival (OS) in SCC cases with positive PD‐L1 expression in comparison to cases who had marginal PD‐L1 expression. DFS was notably worse in AC cases who had PD‐L1‐positive macrophages compared to AC patients with no PD‐L1‐positive macrophages.

Other studies have also assessed PD‐L1 expression in CC. In a retrospective study, Omenai et al.^15^ evaluated the expression of PD‐L1 in CC in 183 paraffin‐embedded and formalin‐fixed tissue blocks. Also, 57.4% of CC samples were PD‐L1 positive. Comparing histological subtypes, PD‐L1 was expressed in 58.7% vs 50% in SCC and AC, respectively similar to our study, a higher incidence of PD‐L1 expression was reported in SCC rather than AC. Their study reported that the samples with poorly differentiated CC, adenoid cystic carcinoma, and basaloid SCC, were less likely to express PD‐L1. Similarly, in Reddy et al.'s study,^19^ 32 out of 93 CCs were PD‐L1 positive (34.4%). Among the different subgroups, PD‐L1 was positive in 37.8% of SCC cases (28 out of 74) versus in 28.6% (2 out of 7) of adenosquamous carcinomas vs. 16.7% (2 out of 12) of endocervical adenocarcinomas.

In another study, Yang et al.^17^ examined paraffin samples of patients in two groups with CIN and SCC. They also utilized immunohistochemistry to detect the PD‐1 expression in macrophages and lymphocytes and PD‐L1 expression in tumor cells within the tissue. They reported that increased PD‐1 and PD‐L1 expression was significantly linked to their HPV status, increased degree of CIN, and tumor metastasis. In addition, increased expression of the PD‐1/PD‐L1 pathway was inked to a reduction in pro‐inflammatory cytokine, interferon γ, and an increase in P16INK4a expression. The authors also noted that the PD‐1 and PD‐L1expression, due to the positive correlation with the progression of tumor and metastasis, can be used as clinical prognostic factors to evaluate the prognosis of individual CIN and CC.

In another study evaluating the HPV infection association with PD‐L1 status, Mezache et al.^20^ investigated PD‐L1 expression in cervical intraepithelial neoplasia (CIN) and its correlation with infection by HPV. The presence of PD‐L1 was high in CINs (95%) and in SCC of the cervix (80%). Also, a significant increase was noted in the detection of PD‐L1 in mononuclear cells when we compare SCC to endometrial and ovarian adenocarcinoma. Their research indicated that PD‐L1 is an estimator of cervical HPV infection and increased in CC tissue and surrounding inflammation as compared to other gynecological malignancies.

In Reddy et al.'s study,^19^ 32 out of 93 CCs (34.4%) were PD‐L1 positive. Among the different subgroups, PD‐L1 was positive in 37.8% of SCC cases (28 out of 74) vs in 28.6% (2 out of 7) of adenosquamous carcinomas vs. 16.7% (2 out of 12) of endocervical adenocarcinomas.

It has been shown that immunotherapy strategies targeting the PD‐L1/PD‐1 axis are effective in inhibiting immune evasion by cancer cells.^9^, ^12^ High levels of PD‐L1 expression have been reported in CC (34%–96%).^19^, ^21^ CC, known for its high PD‐L1 expression and other molecular features, has become a focus of immunotherapy research.^22^ These features have led to the integration of immunotherapy into CC trials.

In June 2018, pembrolizumab became the inaugural immunotherapy agent to gain approval from the US Food and Drug Administration for use as a second‐line therapy for cases who have PD‐L1‐positive persistent or metastatic / recurrent CC.^23^ A recent development, the INTERLACE trial, has unveiled that incorporating induction pembrolizumab alongside concurrent standard chemoradiation in locally advanced CC has notably enhanced progression‐free survival (PFS) and overall survival (OS).^24^

Although this current investigation did not reveal statistically significant variances between the groups, the results align with previous research studies,^16^, ^17^, ^18^, ^20^, ^21^ shedding light on the potential role of PD‐L1 in both AC and SCC of the cervix within the Iranian population. This study reinforces the rationale for inhibiting the PD‐L1/PD‐1 immunosuppressive axis as a potential form of immunotherapy for select cases of CC.

Since our study was retrospective and we did not have a thorough database of the examined patients regarding their outcome characteristics as well as HPV virus infection status, we could not evaluate the oncologic outcomes of our patients and the possible correlation of HPV infection with PD‐L1 positivity.

We had some limitations. Firstly, it has been a retrospective study, which inherently carries biases and limitations associated with data collection and analysis. Additionally, the limited patient outcome data and lack of comprehensive information on HPV infection status would restrict our ability to draw definitive conclusions regarding the link between PD‐L1 expression, survival outcomes and HPV infection. Furthermore, our sample size was relatively small, and larger studies should validate our findings and strengthen the statistical power.

Based on our study, we recommend further prospective trials with larger sample sizes and comprehensive patient data to assess the link between PD‐L1 expression, HPV infection status, and survival outcomes in CC. Opting for other molecular features, such as tumor mutational burden and microsatellite instability, would provide a comprehensive understanding of the tumor biology and individualized potential therapeutic agents.

## 5 CONCLUSION

The mean percentage of PD‐L1 expression in the SCC group was 5.19 (SD = 7.1) and 3.44 (SD = 5.7) in the AC group (*p* = .37). We observed more positive cases with positive expression in the SCC group (*p* = .31). We noted a higher, albeit not statistically significant, expression of PD‐L1 in the SCC of the cervix.

## AUTHOR CONTRIBUTIONS

**Maryam Sadat Hosseini:** Data curation (equal); investigation (equal); writing – original draft (equal). **Fatemeh Shafizadeh:** Conceptualization (equal); investigation (equal); writing – original draft (equal). **Mohammad Hashemi Bahremani:** Writing – original draft (equal). **Farah Farzaneh:** Data curation (equal); writing – original draft (equal). **Tahereh Ashrafganjoei:** Methodology (equal); writing – original draft (equal). **Maliheh Arab:** Investigation (equal); writing – original draft (equal); writing – review and editing (equal). **Maryam Talayeh:** Data curation (equal); writing – original draft (equal); writing – review and editing (equal). **Fatemeh Jafari:** Writing – original draft (equal); writing – review and editing (equal). **Alireza Abdshah:** Software (equal); writing – original draft (equal); writing – review and editing (equal).

## CONFLICT OF INTEREST STATEMENT

The authors have stated explicitly that there are no conflicts of interest in connection with this article.

## ETHICS STATEMENT

This research was reviewed by the Ethics Committee of Shahid Beheshti University of Medical Sciences under the code of IR.SBMU.RETECH.REC.1401.639.

## Data Availability

The data used in this study are available from the corresponding author on request.
